# Transmission of Oyster Mushroom Spherical Virus to Progeny via Basidiospores and Horizontally to a New Host *Pleurotus floridanus*

**DOI:** 10.3390/ijms25115677

**Published:** 2024-05-23

**Authors:** Yifan Wang, Zhidong Wen, Yaoyao Yang, Xiangting Hu, Zhizhong Song, Haijing Hu, Guoyue Song, Lunhe You, Jianrui Wang, Yu Liu, Xianhao Cheng, Xiaoyan Zhang

**Affiliations:** 1 School of Agriculture, Ludong University, Yantai 264025, China; wangyifan7798@126.com (Y.W.); haijing975@163.com (H.H.); 19853505259@163.com (G.S.); youlunhe98@163.com (L.Y.); jianrui302@163.com (J.W.); 2713@ldu.edu.cn (Y.L.); chengxianhao@sohu.com (X.C.); 2 Yantai Growth Drivers Conversion Research Institute and Yantai Science and Technology Achievement Transfer and Transformation Demonstration Base, Yantai 264001, China; syj8905@126.com (Z.W.); yyyanghuanghai@126.com (Y.Y.); 17861103082@163.com (X.H.); 3 The Engineering Research Institute of Agriculture and Forestry, Ludong University, No. 186 Hongqizhong Road, Yantai 264025, China; szhzh2000@163.com; 4 Department of Plant Science, University of Cambridge, Cambridge CB2 3EA, UK

**Keywords:** Oyster mushroom spherical virus, vertical transmission, sexual basidiospores, horizontal transmission, *Pleurotus floridanus*

## Abstract

Mycoviruses are usually transmitted horizontally via hyphal anastomosis and vertically through sporulation in natural settings. Oyster mushroom spherical virus (OMSV) is a mycovirus that infects *Pleurotus ostreatus*, with horizontal transmission via hyphal anastomosis. However, whether OMSV can be vertically transmitted is unclear. This study aimed to investigate the transmission characteristics of OMSV to progeny via basidiospores and horizontally to a new host. A total of 37 single-basidiospore offspring were obtained from OMSV-infected *P. ostreatus* and *Pleurotus pulmonarius* for Western blot detection of OMSV. The OMSV-carrying rate among monokaryotic isolates was 19% in *P. ostreatus* and 44% in *P. pulmonarius*. Then, OMSV-free and OMSV-infected monokaryotic isolates were selected for hybridization with harvested dikaryotic progeny strains. Western blot analyses of the offspring revealed that the OMSV transmission efficiency was 50% in *P. ostreatus* and 75% in *P. pulmonarius*, indicating vertical transmission via sexual basidiospores. Furthermore, we observed the horizontal transfer of OMSV from *P. pulmonarius* to *Pleurotus floridanus*. OMSV infection in *P. floridanus* resulted in significant inhibition of mycelial growth and yield loss. This study was novel in reporting the vertical transmission of OMSV through basidiospores, and its infection and pathogenicity in a new host *P. floridanus*.

## 1. Introduction

Mycoviruses are prevalent in fungi, encompassing yeasts, macrofungi, and various pathogenic fungi that infect plants, insects, and humans. The first identification of a mycovirus occurred in 1962 with the discovery of the La France isometric virus (LIV) in the economically significant mushroom species *Agaricus bisporus* [1,2]. Since then, approximately 60 mycoviruses infecting edible fungi have been recorded, comprising double-stranded (ds)RNA, positive-sense single-stranded (+)ssRNA, and negative-sense single-stranded (−)ssRNA viruses [3,4]. Although most mycoviruses establish latent or symptomless infections within their fungal hosts, some can cause severe symptoms such as changes in growth, colony morphology, spore production, pigmentation, and pathogenicity [1,5,6,7].

In nature, mycoviruses are primarily transmitted horizontally via hyphal fusion and vertically through sporulation [8]. Vertical transmission occurs mainly via the passage of asexual and sexual spores from parents to offspring, with transmission efficiency varying among different fungal viruses [9,10]. For example, the (+)ssRNA Cryphonectria hypovirus 1 (CHV1) exhibited a 100% transmission rate to conidiospore progeny, albeit only 50% of the offspring showed virulence in the offspring of sexual spores [11]. Similarly, the dsRNA *Fusarium graminearum* hypovirus 1 (FgHV1) infecting *F. graminearum* can be transmitted vertically through asexual spores with a 100% transmission rate [12]. Another dsRNA *Rhizoctonia solani* partitivirus (RsPV-BS5) can exist stably in the sexual spores of *R. solani* AG-3, indicating that RsPV-BS5 can carry out vertical transmission in *R. solani* [13]. However, few studies exist about the vertical transmission of mycoviruses within edible mushroom species. In *A. bisporus*, the LIV has an average carrier rate of 65–75% within basidiospores [14]. In *Lentinula edodes*, *L. edodes* negative-stranded RNA virus 1 (LeNSRV1) is transmitted through basidiospores, albeit with relatively low efficiency [15]. *L. edodes* spherical virus (LeSV) is a dsRNA mycovirus infecting *L. edodes*, exhibiting a 90% LeSV-carrying rate in basidiospores [16]. To date, no studies have investigated the vertical transmission involving (+)ssRNA viruses in edible mushroom species.

Horizontal transmission via mycelium serves as a major means of mycovirus spread, with mycoviruses usually exhibiting a restricted host range, infecting the same or closely affiliated compatible groups [1,7,17]. For example, RsPV-BS5 can be transmitted horizontally from the virus-infected strain BS-5 to the virus-free strain 06-2-15 of *R. solani* [13]. Through co-infection experiments, *Beauveria bassiana* chrysovirus 2 (BbCV2) was horizontally transmitted from the virus-infected strain BbOFZK152 to the virus-free strain BbOFDH1-5-GFP [18]. Additionally, *Cryphonectria naterciae* fusagravirus 1 (CnFGV1) exhibited the capacity for transmissibility to other *Cryphonectria* species [19]. Of the ten *Cryphonectria* species isolates tested, six were successfully infected by CHV1 from *C. parasitica* via hyphal anastomosis [20]. In *A. bisporus*, mushroom virus X (MVX) was demonstrated to be transmitted horizontally via hyphal anastomosis from an MVX-infected strain to five other MVX-free strains [21]. Additionally, the horizontal transmission of *Cordyceps chanhua* partitivirus 1 (CchPV1) was observed in *C. chanhua*, spreading from strain RCEF5997 to RCEF5833 [22].

OMSV was first reported infecting *P. ostreatus* mushrooms in Korea in 2003, which was closely related to the mushroom die-back disease. It is a spherical virion with a diameter of 27 nm, harboring a (+)ssRNA genome. The complete genome of OMSV contains 5784 nucleotides encoding 7 open reading frames, with ORF1 encoding the RNA-dependent RNA polymerase and ORF2 encoding the coat protein (CP). However, the functions of the proteins predicted to be encoded by ORF3-7 currently remain unidentified [23]. OMSV occurrence has been reported in several regions in China, including Jilin province, Shandong province, and Beijing city [24,25,26]. Previous studies found that OMSV could be transmitted horizontally from an infected strain to a virus-cured strain by hyphae in *P. ostreatus* strain 8129, resulting in slowed mycelial growth, malformations of the fruiting bodies, and yield loss [26]. Interestingly, the OMSV could also cross the species barrier and horizontally transmit from *P. ostreatus* to *P. pulmonarius* [27]. However, no studies have investigated the vertical transmission of OMSV.

This study aimed to investigate whether OMSV could be transmitted vertically through sexual basidiospores and whether it posed a threat to other *Pleurotus* species through horizontal transmission. This study demonstrated that OMSV could be transmitted to offspring via basidiospores, making this study novel in reporting the vertical transmission of OMSV. In addition, the horizontal transmission of OMSV from *P. pulmonarius* to *P. floridanus* was observed. The OMSV infection not only reduced the mycelial growth rate but also led to a decrease in the yield of fruiting bodies in *P. floridanus*. This study provided a solid foundation for developing virus-free and antiviral strains, thereby contributing to the sustainable growth of the *P. ostreatus* industry.

## 2. Results

### 2.1. Transmission of OMSV by Single Spores

**Figure 1:**
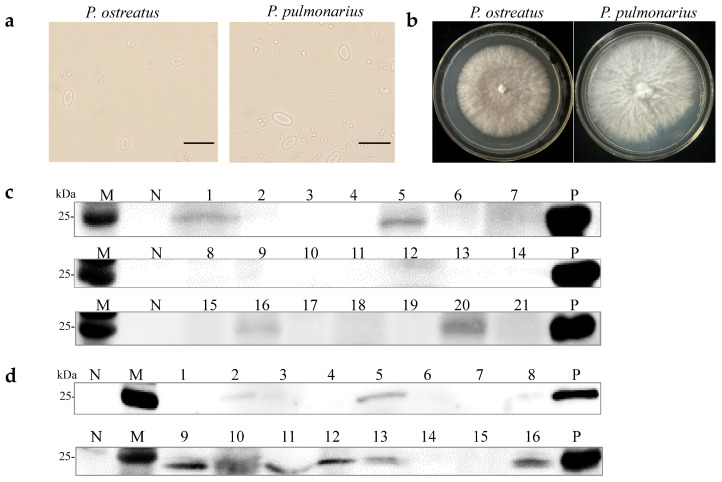
Detection of OMSV for monokaryotic strains of *P. ostreatus* and *P. pulmonarius*. (**a**) Microscopic morphology of basidiospores of *P. ostreatus* or *P. pulmonarius*. Scale bars = 10 µm. (**b**) Subculturing of selected monokaryotic progeny strains from the colonies of *P. ostreatus* or *P. pulmonarius*. Western blot detection of OMSV in 21 *P. ostreatus* monokaryotic strains (**c**) and 16 *P. pulmonarius* monokaryotic strains (**d**). OMSV-free and OMSV-infected strains served as negative (N) and positive (P) controls, respectively. Lane M, molecular weights of protein marker.

A previous study demonstrated that OMSV could be horizontally transmitted via mycelia [27]. Two OMSV-infected strains, *P. ostreatus* strain 8129 and *P. pulmonarius* strain XH2208, were cultivated to investigate the possibility of vertical transmission through sexual basidiospores. Basidiospores from both *P. ostreatus* and *P. pulmonarius* were isolated from the basidiocarp and cultured on potato dextrose agar (PDA) plates (Figure 1a). Further, 21 basidiospores of *P. ostreatus* and 16 basidiospores of *P. pulmonarius* were collected and germinated, resulting in the development of monokaryotic colonies within 10 days (Figure 1b). Optical microscopy was employed to confirm the monokaryotic strains based on the absence of clamp connections, followed by the further detection of OMSV. Western blot analyses revealed that 19% of *P. ostreatus* monokaryotic strains (4/21) carried OMSV (Figure 1c, Lanes 1, 5, 16, and 20), whereas 44% of *P. pulmonarius* monokaryotic strains (7/16) carried OMSV (Figure 1d, Lanes 2, 5, 9, 11, 12, 13, and 16). These findings indicated that OMSV could persistently exist within the sexual basidiospores of both *P. ostreatus* strain 8129 and *P. pulmonarius* strain XH2208.

### 2.2. Transmission of OMSV from Single Basidiospores to Dikaryotic Strain after Single-Spore Hybridization

**Figure 2:**
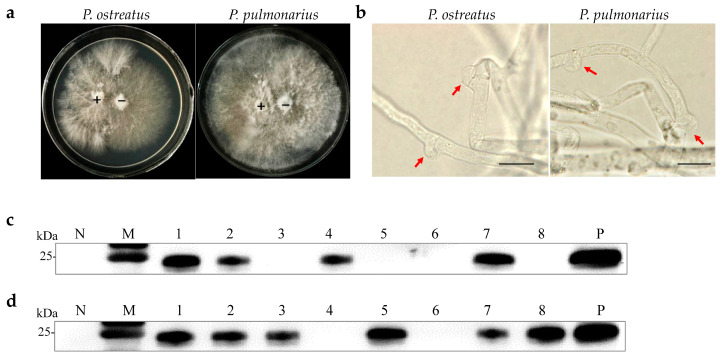
OMSV detection in dikaryotic progeny strains of *P. ostreatus* and *P. pulmonarius*. (**a**) Dual culture of OMSV-infected (+) and OMSV-free (–) monokaryotic isolates. (**b**) Microscopic morphology of clamp connection. The red arrow indicates the clamp connection. Scale bars = 10 µm. Western blot analysis of OMSV in dikaryotic progeny strains of *P. ostreatus* (**c**) and *P. pulmonarius* (**d**); OMSV-free and OMSV-infected strains served as negative (N) and positive (P) controls, respectively. Lane M, molecular weights of protein marker.

Monosporous hybridization is a vital approach in breeding *Pleurotus* species cultivars. Six OMSV-free and four OMSV-infected *P. ostreatus* monokaryotic isolates were selected and cultured equidistantly for co-cultivation to investigate whether OMSV could be transmitted to offspring through monosporous hybridization. Similarly, four OMSV-free and seven OMSV-infected *P. pulmonarius* monokaryotic isolates were selected and cultured equidistantly for co-cultivation. Each OMSV-infected monokaryotic isolate was crossed with each OMSV-free monokaryotic isolate in all possible combinations, resulting in 24 *P. ostreatus* crosses and 28 *P. pulmonarius* crosses. After 14 days of incubation, dikaryotic progeny strains were formed at both ends of the anastomosis where the hyphae of the two monokaryotic isolates met. The progeny strains underwent subculture on PDA (Figure 2a), followed by the microscopic examination of clamp connections to confirm the presence of dikaryotic hyphae (Figure 2b). The presence of clamp connection indicated the successful mating of these cross combinations. A total of eight successful *P. ostreatus* crosses and eight *P. pulmonarius* crosses were identified, and then the sixteen dikaryotic progeny strains were subjected to Western blot detection of OMSV. The results revealed that 50% of *P. ostreatus* dikaryotic progeny strains (4/8) carried OMSV (Figure 2c) whereas 75% of *P. pulmonarius* dikaryotic progeny strains (6/8) carried OMSV (Figure 2d).

### 2.3. Infection of the New Host P. floridanus by OMSV across the Species Barrier

**Figure 3:**
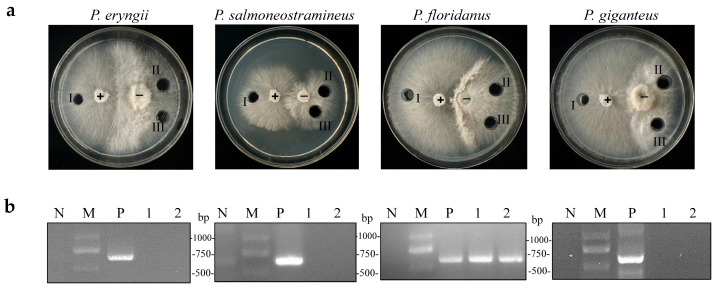
Co-cultivation of OMSV-positive (+) *P*. *pulmonarius* and OMSV-free (−) *Pleurotus* spp. (**a**) Co-cultivation of the donor strain with *P. eryngii*, *P. salmoneostramineus*, *P. floridanus*, and *P. giganteus*. One inoculum from the donor side (I) and two inocula from the recipient (II and III) were sub-cultivated for further detection of OMSV. (**b**) RT-PCR detection of OMSV in different inoculants. OMSV-free strains of *P. eryngii* C1021, *P. salmoneostramineus* TH20901, *P. floridanus* LDPF2305 or *P. giganteus* ZD2308 served as negative controls (N). M, DNA Marker2000.

A previous study discovered that OMSV could cross the species barrier and infect *P. pulmonarius* [23]. An OMSV-infected *P. pulmonarius* strain was employed as a donor, which was co-cultivated with OMSV-free strains of *P. eryngii*, *P. salmoneostramineus*, *P. floridanus*, and *P. giganteus*, to investigate whether OMSV could infect other *Pleurotus* species. After several days of co-cultivation, an antagonistic relationship was observed between the donor and recipient strains (Figure 3a). Subsequently, an inoculum derived from the donor culture and two inoculums from the recipient culture were sub-cultured for 7 days. Reverse-transcription (RT) polymerase chain reaction was then employed to confirm the presence or absence of OMSV within all the tested strains. *P. floridanus* tested positive for OMSV, whereas the other three *Pleurotus* species remained negative under identical conditions (Figure 3b). Therefore, this result suggested a successful transfer of OMSV from *P. pulmonarius* to *P. floridanus.*

### 2.4. Slowing down of the Mycelial Growth of P. floridanus by OMSV Infection

**Figure 4:**
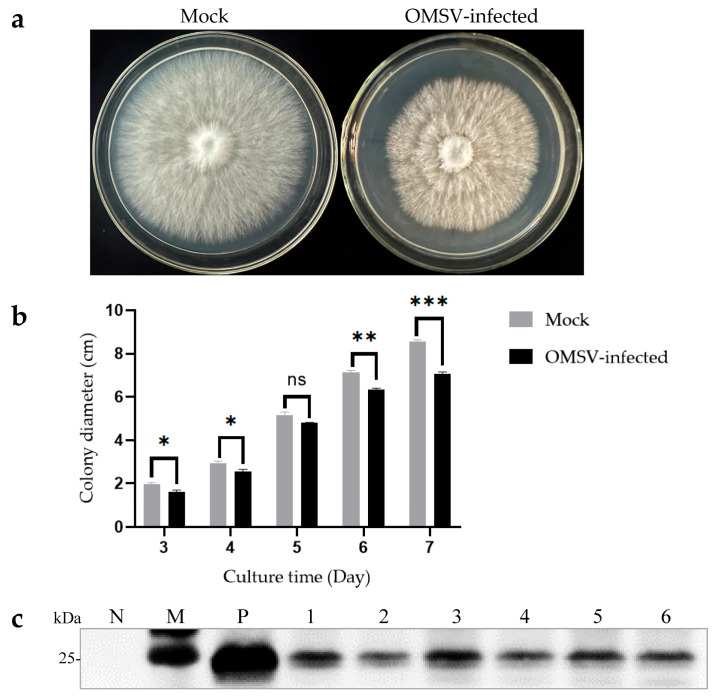
Morphological characteristics of OMSV-free and OMSV-infected strains of *Pleurotus floridanus* following 7 days of incubation. Colony morphology (**a**) and growth rate (**b**) of the hyphae of OMSV-infected and OMSV-free (mock) strains of *P. floridanus*. (**c**) Western blot analysis of OMSV in *P. floridanus* fruiting bodies. Samples 1–6 correspond to six biological replicates. The OMSV-free *P. floridanus* sample served as negative control (N) and OMSV-infected *P. pulmonarius* served as positive control (P); Lane M, molecular weights of protein marker. The independent sample *t*-test was performed between the OMSV-free and OMSV-infected isogenic strains (ns, not significant; * *p* < 0.05; ** *p* < 0.01; *** *p* < 0.001).

The isogenic strains that were OMSV-free and OMSV-infected were cultured on PDA plates to investigate the effect of OMSV infection on the growth of *P. floridanus* mycelium (Figure 4a). After 7 days of incubation, the OMSV-infected strain showed a significantly slower mycelial growth compared with the OMSV-free strain (Figure 4b). To confirm this observation, the mycelial growth rate was monitored by measuring colony diameters every day for 1 week. On day 7 after inoculation, the OMSV-infected strain exhibited a growth rate approximately 0.83-fold that of the OMSV-free strain.

### 2.5. A Reduction in Fruiting Body Yield of P. floridanus by OMSV Infection

Cultivation experiments were conducted using both OMSV-free and OMSV-infected isogenic strains to explore the potential impact of OMSV infection on the fruiting bodies of *P. floridanus*. The average time it took for the first flush of mushrooms to be harvested from inoculation was calculated. Interestingly, the OMSV-infected strain required an additional 4 days for harvest relative to the OMSV-free strain, indicating a deceleration in mycelial growth during culture (Table 1). To evaluate yield, both the first and second flushes of fruiting bodies were harvested and their average yield was calculated (Table 1). Significant differences were observed between the yields of the OMSV-free and OMSV-infected strains in the first flush. The average yield per bag for cultivated fruiting bodies in the OMSV-free strain was 194.08 g/bag, representing a 1.36-fold increase compared with that of its infected counterpart (141.94 g/bag). Upon reaching the second flush, the average yield per bag for the OMSV-infected strain decreased to 126.98 g/bag, representing a reduction by 0.72-fold compared with that of its uninfected counterpart (176.97 g/bag) (Table 1). The findings strongly indicated that OMSV infection led to a decrease in fruiting body yield in *P. floridanus*. Deformed fruiting bodies were collected and Western blot analysis was performed to confirm whether OMSV existed in these fruiting bodies. The results revealed the presence of OMSV-CP in the fruiting bodies of *P. floridanus* (Figure 4c).

### 2.6. Vertical Spread of OMSV in the New Host

**Figure 5:**
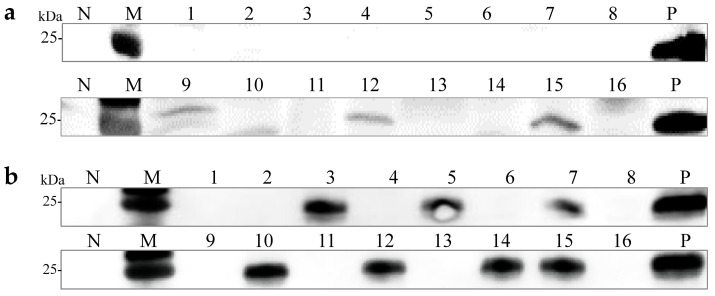
Vertical transmission of OMSV in *P. floridanus*. (**a**) Western blot analysis confirmed OMSV infection in *P. floridanus* monokaryotic strains. (**b**) Western blot analysis of OMSV detection in *P. floridanus* dikaryotic progeny strains. The healthy *P. floridanus* sample served as negative control (N). Lane M, molecular weights of protein marker.

Single spores of *P. floridanus* were collected and the monokaryotic strains formed after single-spore culture were examined to assess the vertical transmission potential of OMSV via basidiospores in a new host. A total of 16 spores were randomly selected for Western blot analysis, revealing that 19% of *P. floridanus* monokaryotic strains (3/16) carried OMSV (Figure 5a). Thirteen OMSV-free and three OMSV-infected *P. floridanus* monokaryotic isolates were selected and cultured at equidistance for co-cultivation. Each OMSV-infected monokaryotic isolate was crossed with each OMSV-free monokaryotic isolate in all possible combinations, resulting in 39 distinct *P. floridanus* crosses. After 14 days of incubation, dikaryotic progeny strains were observed at both ends of the anastomosis where the hyphae of the two monokaryotic isolates met, and these dikaryotic progeny strains were sub-cultured. Then, 16 successful hybridization groups were identified, and the dikaryotic strains were examined using Western blot analysis, revealing that 44% of *P. floridanus* dikaryotic progeny strains (7/16) carried OMSV (Figure 5b).

## 3. Discussion

Mycoviruses typically spread vertically through sporulation [8] but the efficiency varies among different fungal viruses [10]. For instance, CHV1 can be transmitted to conidiospore offspring with a 100% transmission rate but maintains only 50% virulence in sexual spores offspring [11]. Similarly, RsPV-BS5 was found in *R. solani* basidiospores, confirming its vertical transmission [13]. In *L. edodes*, the prevalence rate of LeSV in basidiospores was 90% [16]. However, investigations into the vertical transmission of mycoviruses in *P. ostreatus* are limited. This study demonstrated, for the first time, the vertical transmission of OMSV via basidiospores in *P. ostreatus*, *P. pulmonarius*, and *P. floridanus*. Monokaryotic strains of these *Pleurotus* species were found to carry OMSV, with carrying rates of 19%, 44%, and 19%, respectively. This suggested the vertical transmission of OMSV through sexual basidiospores. Based on these results, the transmission of OMSV to offspring through single-spore hybridization was explored. In the present study, dikaryotic progeny strains of *P. ostreatus*, *P. pulmonarius*, and *P. floridanus* were found to carry OMSV, with carrying rates of 50%, 75%, and 44%, respectively. Overall, this indicated the potential of OMSV to transfer vertically through successive generations within its host organism. This study was novel in employing single-spore hybridization technology to explore the vertical transmission of OMSV. Studies have reported that the vertical transmission efficiency of fungal viruses is related to the age of mycelia [28,29,30,31]. Based on it, we will explore the effect of mycelium age on the vertical transmission of OMSV. Considering the importance of single-spore hybridization in *Pleurotus* breeding, an OMSV-free parental strain is the first choice in cultivating virus-free cultivars. As for the parent strains that carry OMSV, we can screen the OMSV-free single spores for hybridization to obtain OMSV-free strains.

The horizontal transmission of mycoviruses occurs through mycelia. The *Leptosphaeria biglobosa* botybirnavirus 1 can cross-species transmit from *Leptosphaeria biglobosa* to *Botrytis cinerea* [32]. The *Cryphonectria nitschkei* chrysovirus 1 can be horizontally transmitted from its original host *C. nitschkei* to *C. radicalis* and *C. naterciae* strains [33]. A previous study conducted by the authors confirmed the horizontal transmission capability of the OMSV Chinese strain (OMSV-Ch) from *P. ostreatus* 8129 to *P. pulmonarius* strain XH2208 via mycelial contact [27]. In this study, OMSV-infected *P. pulmonarius* was co-cultured with other strains of *Pleurotus* species, revealing that OMSV could indeed spread horizontally from *P. pulmonarius* to *P. floridanus*. However, attempts to transmit OMSV to *P. eryngii*, *P. salmoneostramineus*, and *P. giganteus* were unsuccessful, likely due to a robust transmission barrier between the test strains. In addition, phylogenetic analyses suggested a closer evolutionary relationship between *P. pulmonarius* and *P. floridanus* but indicated a distant evolutionary relationship with *P. eryngii*, *P. giganteus*, and *P. salmoneostramineus* [34]. This implied the existence of a more formidable interspecies transmission barrier between these two non-transmissible species. In order to understand the interaction between OMSV and fungi, it is necessary for us to explore the molecular mechanism of OMSV movement in *Pleurotus* species. The OMSV-infected *P. floridanus* can be used as a donor strain to study whether the OMSV can infect the other OMSV-free *Pleurotus* species, which will help us understand the mechanisms underlying OMSV’s horizontal transmission.

Mycovirus infections often manifest with subtle symptoms or asymptomatically, but some can significantly affect fungal hosts [6]. The LIV has been implicated in causing mycelial degeneration and substantial yield reductions in *A. biporus* [35]. The MVX can destroy the primordium, leading to premature opening, discoloration, and malformed fruiting bodies of *A. biporus* [21,36]. In *L. edodes*, LePV1 has been associated with manifestations of both mycelial degradation and abnormal fruiting body formation [37]. The *Pleurotus ostreatus* ASI2792 mycovirus (PoV-ASI2792) has been reported to slow down mycelial growth and cause yield loss in the fruiting bodies of *P. ostreatus* [38]. More recently, it was shown that OMSV-Ch inhibited mycelial growth, induced dysmorphic symptoms in fruiting bodies, and reduced mushroom yield in both *P. ostreatus* and *P. pulmonarius* [27]. In the present study, the OMSV infection in *P. floridanus* resulted in a slowdown of mycelial growth and a decrease in mushroom yields. Thus, across three different *Pleurotus* species, OMSV emerged as a likely primary causative agent of yield losses in the host. Although the exact mechanisms underlying OMSV pathogenicity remain unclear, Previously, it was reported that the infection of PoV in *P. ostreatus* affects mycelial growth and fruiting body formation by directly reducing gene expression and then impairing the activity of some extracellular enzymes [38]. The OMSV infection reduced the carboxymethyl cellulase and laccase activity in the *P. pulmonarius* strain [27]. Further studies concerning the relative expression levels of genes and the activity levels of other extracellular enzymes such as amylase, aspartic protease, cellulase, chitinase, β-glucosidase, lipase, manganese peroxidases, polygalacturonase, and xylanase are necessary to investigate. This exploration aimed to better understand the potential molecular underpinnings of the detrimental effects of OMSV infection.

## 4. Materials and Methods

### 4.1. Single-Spore Isolation of P. ostreatus and P. pulmonarius

To collect basidiospores from the OMSV-infected mature fruiting bodies of *P. floridanus* strain LDPF2305, *P. ostreatus* strain 8129, and *P. pulmonarius* strain XH2208, the stipes were removed. All the tested strains were derived from the Fungarium of Ludong University. The pileus was gently positioned, with the gills facing down, on a sterilized Petri dish to allow basidiospores to naturally shed from the gills and settle onto the dish surface, thereby forming a spore print. Subsequently, the basidiospores were carefully harvested and mixed with sterile water to prepare a spore suspension. The basidiospore count was precisely determined using a hemocytometer (Shanghai Qiujing Biochemical Rechemical Reagent and Instrument Co., Ltd., Shanghai, China), and spore suspension was accordingly adjusted to a concentration of 100 basidiospores/mL. A volume of 100 μL of the diluted basidiospore suspension was evenly spread onto PDA to isolate 20–30 monokaryotic strains. The inoculated plates were then incubated at a constant temperature of 24 °C for 10 days until colonies of approximately 1 cm in diameter emerged from germinating basidiospores. The microscopic examination confirmed the absence of clamp connections.

### 4.2. Monosporous Hybridization

Monosporous hybridization was performed using the two-point method. A dual-culture technique was employed to facilitate the anastomosis of monokaryotic isolates from two strains each of *P. floridanus*, *P. ostreatus*, and *P. pulmonarius*, encompassing all feasible pairings. An OMSV-free monokaryotic isolate and an OMSV-infected monokaryotic isolate were inoculated on opposite sides of a PDA, maintaining a 10 mm distance between them. The plate was then incubated at a constant temperature of 25 °C. Dikaryotic progeny strains were formed at both ends of the anastomosis where the hyphae of the two monokaryotic isolates met, typically requiring approximately 14 days. The dikaryotic progeny strains were sub-cultured on PDA. Successful hybridization was confirmed if clamp connection structures were observed under the microscope.

### 4.3. Protein Extraction and Western Blot

The hyphae to be detected was ground into powder with liquid nitrogen, followed by thorough mixing with 2 × SDS loading buffer [27]. This mixture was subjected to boiling at a temperature of 100 °C for 10 min, and then it was centrifuged for 10 min at 12,000 rpm. The samples were separated by electrophoresis via 12.5% SDS-PAGE gels and subsequently transferred onto a nitrocellulose membrane (GE Healthcare). For detection of OMSV, the membrane was subjected to incubation with polyclonal antibodies against OMSV CP, followed by treatment with horseradish peroxidase-conjugated goat anti-rabbit secondary antibodies [27]. The detection of antibody–antigen binding interactions was visualized by using Omni-ECL™ Pico Light Chemiluminescence Kit. The secondary antibody and chemiluminescence kit were sourced from Shanghai Epizyme Biomedical Technology (Shanghai, China).

### 4.4. Horizontal Transmission of OMSV

Horizontal transmission experiments were executed using OMSV-infected *P. pulmonarius* as a donor strain. OMSV-infected strain (*P. pulmonarius* strain XH2208) and OMSV-free strain (*P. floridanus* strain LDPF2305, *P. eryngii* strain C1021, *P. salmoneostramineus* strain TH20901, and *P. giganteus* strain ZD2308) were co-cultivated separately on PDA at a constant temperature of 25 °C for a duration of 7 days, and all strains were from the Fungarium of the Ludong University. Upon completion of the co-culture, three mycelial agar plugs (I, II, and III) were selected for sub-culture on PDA. The occurrence of horizontal transmission of OMSV was subsequently detected through RT-PCR amplification, utilizing OMSV-specific primer.

### 4.5. RNA Extraction and Reverse Transcription PCR (RT-PCR)

Approximately 0.1 g fresh mycelium was collected and homogenized in liquid nitrogen. Total RNA extraction was accomplished using RNA Easy Fast Plant Tissue Kit (Tiangen, Beijing, China). To perform the reverse transcription reaction, 10 μL RT master mixture was prepared, comprising 5 μL ddH_2_O, 3 μL 5 × RT-PCR buffer, 1 μL dNTP mix, 0.5 μL reverse primer (OMSV-R, GAGATGTAGACRTTGAAAGC), 0.25 μL M-MLV reverse transcriptase, and 0.25 μL RNase Inhibitor, which was then combined with 5 μL of RNA. Reverse transcription proceeded at 37 °C for a duration of 90 min. Following the RT reaction, 30 μL PCR mixture containing 15 µL 2 × Taq PCR MasterMixII (Tiangen, Beijing, China), 2 µL cDNA template, 1 µL of specific primers (OMSV-F/OMSV-R, ACCCCCCCAGGATCTCAAGCTTC/GAGATGTAGACRTTGAAAGC), and 11 µL ddH_2_O [26]. The resulting PCR amplicons were subjected to electrophoretic separation in a 1% agarose gel, operating at a voltage of 120 V for 45 min.

### 4.6. Mycelial Growth Rate Measurement

To investigate the influence of OMSV on mycelial growth in *P. floridanus*, OMSV-free and OMSV-infected strains were individually inoculated at the central point of PDA (9 cm in diameter), which were incubated in darkness at 25 °C. Following inoculation, the vertical line was made with the center of the mycelium block as the intersection point. It took about 7 days to record growth length of mycelium every day until the colony diameters filled the plate. Concurrently, the average mycelium growth rate (mm/day) was calculated. All samples were repeated in triplicate.

### 4.7. Cultivation of P. floridanus

A 7.5 mm activated mycelium was cut with a sterilized cylindrical cutter and placed in a 200 mL potato glucose broth (PDB). The culture was maintained in darkness at 25 °C with agitation at 150 rpm for a 10-day period. The basic cultivation substrate consisted of 78% cottonseed shells, 20% wheat bran, and 2% pulverized lime with 60% water saturation and was packed in polypropylene bags (weighing 1.5 kg). Before the substrate mixing, cottonseed shells were immersed in room temperature tap water for 12 h. The moisture content was verified by manually squeezing the substrate until no further water exuded. The blended substrate was sterilized at 121 °C for 3 h and then cooled to 20 °C. Each bag was inoculated with 20 mL *P. floridanus* mycelium which had been previously cultivated in PDB. The bags were then placed in a dark cultivation chamber maintained at 24 °C. When the mycelium filled the whole bag, the bag was transferred to the fruiting room chamber for culture. The bags were carefully opened by making a small incision with a knife. The growth chamber was aerated by periodically opening the door and maintained a relative humidity of 80–85% by humidifier, while the temperature was controlled at 22–24 °C via an air conditioner. The fully mature fruiting bodies were harvested 7 days after the formation of the primordium. During the cropping period, the mushroom flushes were harvested twice, with the cumulative yield from 60 bags of *P. floridanus* being documented. Statistical analysis was performed using SPSS (version 26) software. The independent sample *t*-test was employed to determine the statistical significance of the variations. A *p*-value < 0.05 was employed for defining a statistically significant difference. Data were reported as mean ± standard deviations.

## 5. Conclusions

In summary, this study provided strong evidence of OMSV transmission through sexual basidiospores, demonstrating its vertical transmission capability. Furthermore, OMSV was consistently transmitted to progeny via single-spore hybridization, marking the first reported instance of the vertical transmission of OMSV. Meanwhile, OMSV was found to be horizontally transmitted from *P. pulmonarius* to *P. floridanus*, resulting in significant reductions in mycelial growth and mushroom yield. These findings enhanced the understanding of the potential threats posed by OMSV to *Pleurotus* species.

## Figures and Tables

**Table 1:** Impact of OMSV infection on *Pleurotus floridanus* measured in cultivation tests.

Strains	Period from Inoculation to Harvest (Day)	1st Flush Yield (g/bag)	2nd Flush Yield (g/bag)
OMSV-free	36.00 ± 1.00	194.08 ± 10.13	176.97 ± 12.39
OMSV-infected	40.00 ± 1.00	141.94 ± 8.19 ***	126.98 ± 10.71 *

* *p* < 0.05, Statistical significance; a highly significant difference. *** *p* < 0.001, an extremely significant difference between the OMSV-free and OMSV-infected strains.

## Data Availability

The data presented in this study are available within the article.
